# Release of ranibizumab using a porous poly(dimethylsiloxane) capsule suppressed laser-induced choroidal neovascularization via the transscleral route

**DOI:** 10.1007/s10856-022-06705-z

**Published:** 2022-12-31

**Authors:** Nobuhiro Nagai, Reiko Daigaku, Remi Motoyama, Hirokazu Kaji, Toshiaki Abe

**Affiliations:** 1 Division of Clinical Cell Therapy, United Centers for Advanced Research and Translational Medicine (ART), Tohoku University Graduate School of Medicine, 2-1 Seiryo-machi, Aoba-ku, Sendai, 980-8575 Japan; 2 Department of Biomechanics, Institute of Biomaterials and Bioengineering, Tokyo Medical and Dental University, 2-3-10 Kanda-Surugadai, Chiyoda, Tokyo, 101-0062 Japan

**Keywords:** Drug delivery system, Poly(dimethylsiloxane), Retina, Transscleral delivery, Age-related macular disease, Choroidal neovascularization

## Abstract

The administration of anti-vascular endothelial growth factor drugs in the posterior eye segment with sustained release through less invasive methods is a challenge in the treatment of age-related macular disease. We developed a flexible capsule device using porous poly(dimethylsiloxane) (PDMS) that was able to release ranibizumab. The porous PDMS sheet was fabricated by salt-leaching of a micro-sectioned PDMS sheet containing salt microparticles. Observation with scanning electron microscopy revealed that the pore densities could be adjusted by the concentration of salt. The in vitro release study showed that the release rate of fluorescein isothiocyanate-tagged albumin could be adjusted based on the pore density of the porous PDMS sheet. Ranibizumab could be released in a sustained-release manner for 16 weeks. The device was implanted on the sclera; its efficacy in terms of the suppression of laser-induced choroidal neovascularization (CNV) in rats was compared with that of monthly intravitreal injections of ranibizumab. At 8 and 18 weeks after implantation, the CNV area was significantly reduced in rats that received the ranibizumab-releasing device compared with those that received the placebo device. However, although monthly intravitreal injections of ranibizumab reduced CNV for 8 weeks, this reduction was not sustained for 18 weeks. In conclusion, we demonstrated a novel controlled-release device using a porous PDMS sheet that could suppress CNV via a less invasive transscleral route versus intravitreal injections. This device may also reduce the occurrence of side effects associated with frequent intravitreal injections.

**Graphical Abstract:**

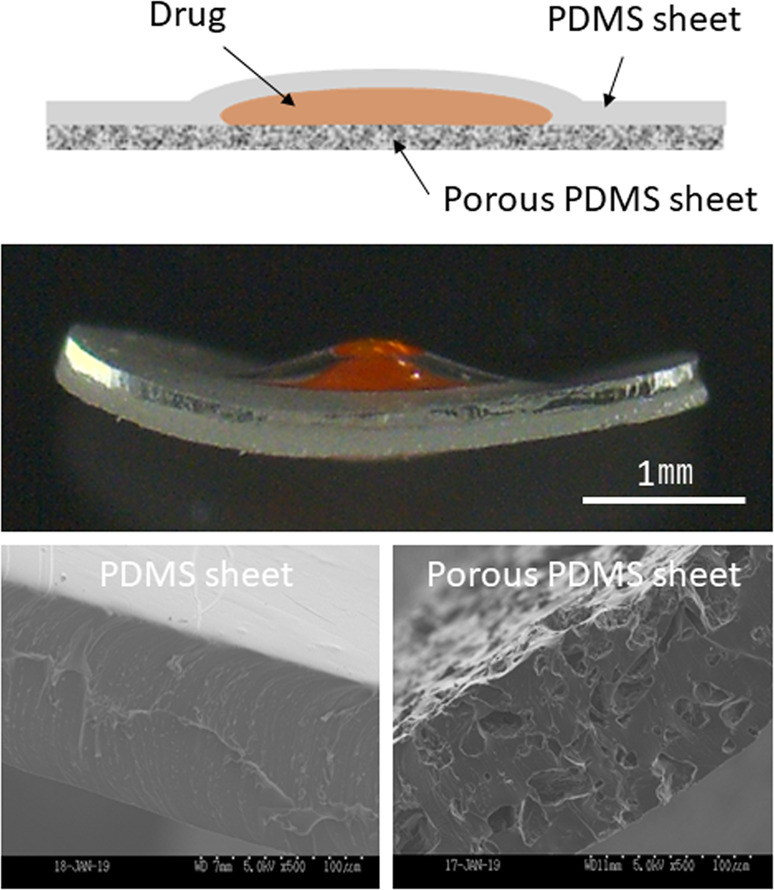

## Introduction

Age-related macular disease (AMD) is characterized by progressive degeneration of photoreceptors and retinal pigment epithelium (RPE) in the macular region of the retina [1]. Wet AMD is a serious form of the disease, and 90% of cases progress to legal blindness [2]. It is characterized by choroidal neovascularization (CNV) as a result of abnormal choroidal blood vessel growth through Bruch’s membrane into the macula [3]. Vascular endothelial growth factor (VEGF) has been identified as a major promoter of the development of CNV. Thus, anti-VEGF therapy has drastically improved visual outcomes and become a mainstay treatment for neovascular retinal diseases [4].

The most common route of delivery to the posterior segment of the eye is through intravitreal injections. Intravitreal injection of anti-VEGF reagents, such as ranibizumab and aflibercept, can improve visual acuity in some patients. Nevertheless, repeated injections can be unpleasant for the patients and are associated with risk of complications [5]. Thus, numerous drug delivery systems (DDS) that can extend the release of anti-VEGF drugs have been developed to address these limitations [6]. Recently, a port delivery system (PDS) that releases ranibizumab for 6 months was approved by the US Food and Drug Administration for the treatment of wet AMD after obtaining successful results in a phase 3 trial [7]. Although the PDS must be surgically implanted, it requires refilling only once every 6 months. However, most clinically used DDS (including the PDS) are intraocular or intravitreal implants, requiring implantation of the devices into the vitreous or the retina [8]. In terms of invasiveness, transscleral drug delivery using episcleral implants may represent a better approach, because there is no requirement for an ocular incision [9].

The use of DDS implanted in the episcleral region of the eye is rare mainly because of problems related to the availability of the active ingredient in the retina following administration via the transscleral route [10]. Drugs must pass through the sclera, choroid circulation, and RPE barrier to reach the retina [11]. Recently, we showed that use of a sustained-release sheet with ranibizumab suppressed laser-induced CNV in rats [12]. The results indicated that the transscleral administration route could be an alternative to intravitreal injections. In this study, we developed an upgraded version of this sheet-type device that has flexibility for injectable use and controlled porosity for tunable release of ranibizumab. The sheet device is composed of porous poly(dimethylsiloxane) (PDMS) fabricated by leaching salt particles in the sheet. The effectiveness of the device in terms of suppression of laser-induced CNV was evaluated via transscleral administration in rats.

## Materials and methods

### Preparation of the porous PDMS sheet

Sodium chloride granule (Wako, Tokyo, Japan) was milled in a mortar, and the resulting powder was filtered through a membrane filter (352340; pore size: 40 μm; BD Falcon, Tokyo, Japan). The salt powder was mixed with PDMS (Silpot 184 W/C; Dow Corning Toray, Tokyo, Japan) at concentrations of 0.8, 0.5, and 0.1 g/mL. The mixture was cast into a PDMS mold (depth: 2 mm, φ: 14 mm) which is oxidized and silanized to aid in the subsequent release of a PDMS/salt pellet, as previously reported [13]. Moreover, degassing was performed in a vacuum desiccator (Sanplatec, Osaka, Japan) until the disappearance of bubbles in the mixture, followed by curing at 80 °C for 3 h. The PDMS/salt pellet was sliced in a thin sheet (thickness: 120 μm) using a microtome (REM-710; Yamato, Saitama, Japan). The sheets were incubated in water at 37 °C for 2 h to leach the salt out, and the procedure was repeated thrice.

### Preparation of the capsule device

**Fig. 1:**
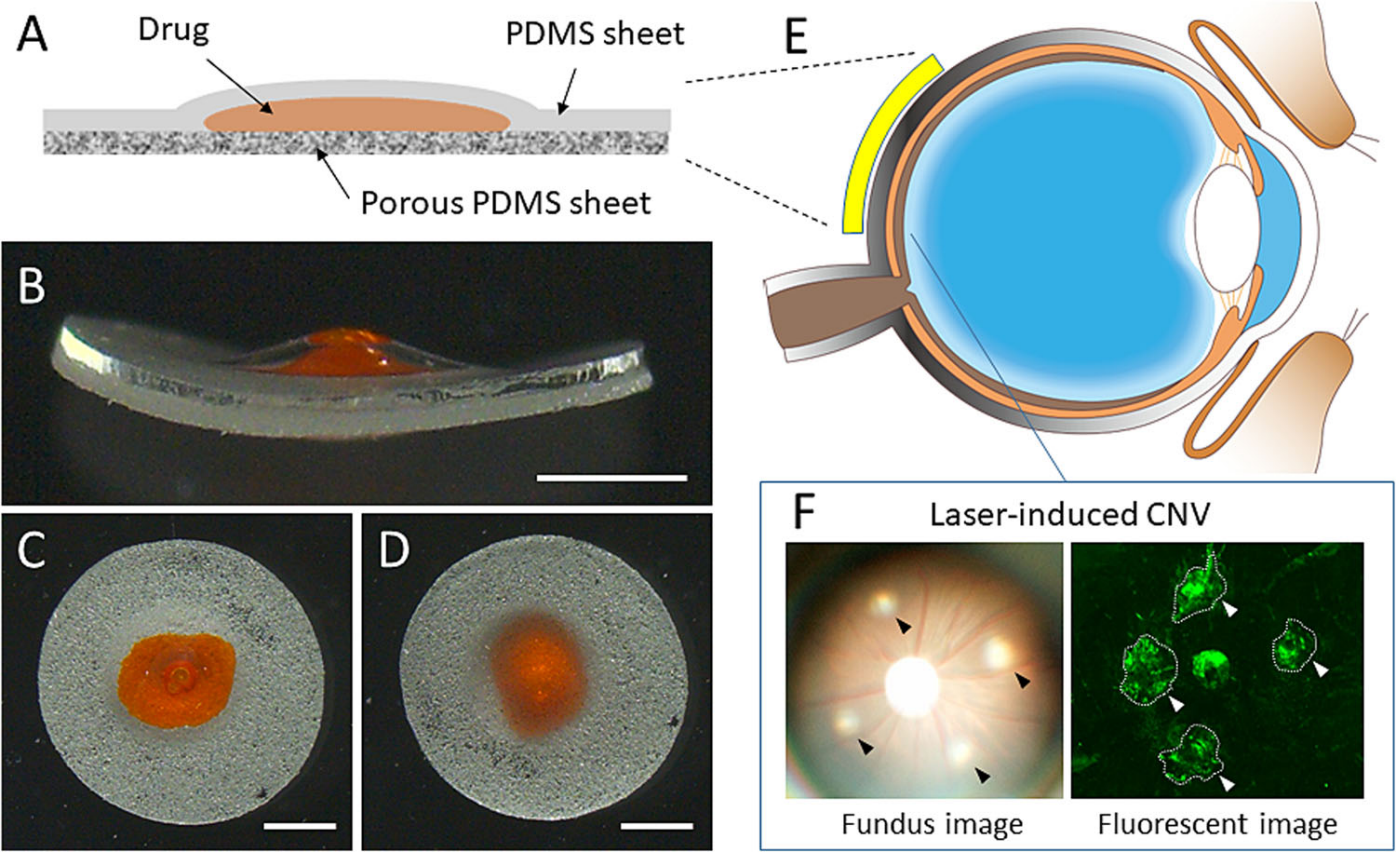
**A** Schematic image of the PDMS sheet capsule device. Photographs of the device from (**B**) the side, (**C**) upper (PDMS sheet), and (**D**) lower (porous PDMS sheet). Schematic image of (**E**) the transscleral drug delivery route and (**F**) laser-induced choroidal neovascularization. Arrowheads show laser burns. Bars: 1 mm. PDMS poly(dimethylsiloxane); CNV choroidal neovascularization

The capsule had a sandwich structure consisting of a porous PDMS sheet, drug formulation, and non-porous PDMS sheet (Fig. 1A). Gelatin from porcine skin (Sigma–Aldrich, Tokyo, Japan) was dissolved at a concentration of 5 wt% in water at 40 °C. Fluorescein isothiocyanate-tagged albumin (FITC-albumin; Sigma–Aldrich) or ranibizumab (Novartis Pharma, Tokyo, Japan) was mixed at a concentration of 50 mg/mL and 5 mg/mL in the gelatin, respectively. The mixtures were filtered using a membrane filter (Millex-HV, 0.45-μm pore size; Millipore, Tokyo, Japan) for sterilization. The mixture (3 μL) was cast onto the center of a porous PDMS sheet and dried for 2 h in a draft chamber. PDMS prepolymer (200 μL) was cast on a non-porous PDMS sheet (thickness: 120 μm). Subsequently, centrifugation at 5,000 rpm for 10 s was performed using a spin-coater (ACT-220DII; Active, Saitama, Japan). The spin-coated non-porous PDMS sheet was preheated at 80 °C for 10 min. The drug-loaded porous PDMS sheet was placed on the spin-coated non-porous PDMS sheet and pressed using PDMS molds. Next, the pressed sheets were heated at 80 °C for 1 h, with the spin-coated PDMS prepolymer acting as an adhesive. The resulting capsule was trimmed in a circular shape (diameter: 4 mm) using a biopsy punch (φ: 4 mm).

### Scanning electron microscopy (SEM)

The porous PDMS sheets were dried in a vacuum dryer (YB-2; Sanplatec, Osaka, Japan), coated with osmium using an osmium coater (HPC-30; Vacuum Device, Ibaraki, Japan), and subjected to SEM. The SEM apparatus (S-3200N; Hitachi, Tokyo, Japan) was operated at 5 kV.

### In vitro release study

The FITC-albumin loaded sheets were incubated in 0.3 mL of phosphate-buffered saline (PBS) at 37 °C. To estimate the amount of FITC-albumin that had diffused out of the sheets, the FITC-albumin content in the PBS solution was measured using a fluorescence plate reader (Infinite F200PRO; Tecan Systems, Tokyo, Japan). The PBS was replaced during the course of the release study to ensure that the concentration of fluorescent molecules was constantly maintained <20% of its saturation value. The results were expressed as the amount determined using a standard curve. In the case of ranibizumab, the ranibizumab-loaded sheets were incubated in 1 mL of PBS at 37 °C. The ranibizumab content in the PBS solution was measured using an anti-VEGF ELISA kit (200-800-AVG; Alpha Diagnostic Intl, Paramus, NJ, USA).

### Animal experiments

Brown Norway rats (SLC, Shizuoka, Japan) (weight: 200–250 g) were used in this study. All animals were handled in accordance with the Association for Research in Vision and Ophthalmology Statement for the Use of Animals in Ophthalmic and Vision Research after receiving approval from the Institutional Animal Care and Use Committee of the Tohoku University Environmental & Safety Committee.

### Treatment

The rats were anesthetized with ketamine hydrochloride (90 mg/kg) and xylazine hydrochloride (10 mg/kg). Their ocular surfaces were anesthetized with a topical instillation of 0.4% oxybuprocaine hydrochloride. A paralimbal conjunctival incision was performed 2.5 mm distant from the temporal limbus, and the sheets were placed onto the left eyes at the sclerae using tweezers. The contralateral eye did not receive treatment.

### CNV procedure

A green argon laser was used to rupture the choroidal membrane using a slit-lamp delivery system (Ultima2000SE; Lumenis, Yokneam, Israel) with a contact lens. The laser settings were as follows: diameter of 50 mm for 0.1 s, at an intensity of 750 mW. Four laser burns were performed around the optic disc. It was confirmed that each burn induced subretinal bubbles, indicating rupture of Bruch’s membrane. CNV laser burns were performed 8–18 weeks after implantation of the ranibizumab and placebo sheets, or intravitreal injections (5 μL in the eye using a Hamilton syringe with a 30-G needle) of ranibizumab solution (10 mg/mL) and PBS (placebo) every 4 weeks.

### Flat mount for CNV area evaluation

The size of the CNV lesion was measured on choroidal flat mounts. Two weeks after performing the CNV laser burns, the rats were perfused with 5 mL of PBS containing 50 mg/mL fluorescein-labeled dextran (FD2000; average Mw: 2,000,000; Sigma–Aldrich) for 3 min. The eyes were enucleated and fixed for 30 min in a fixative solution containing 4% paraformaldehyde. The cornea and lens were removed, and the entire retina was carefully dissected from the eyecup. Four-to-six radial cuts were performed from the edge to the equator, and the eyecup of the RPE-choroid-sclera complex was flat mounted in Permalfuor (Beckman Coulter, Fullerton, CA, USA) with the scleral side facing downward. The flat mounts were examined by fluorescence microscopy (BZ9000; Keyence, Osaka, Japan), and the total area of each CNV zone associated with each burn was measured. The CNV lesions were identified by the presence of fluorescent blood vessels on the choroidal/retinal interface circumscribed by a region lacking fluorescence. Two retina specialists and one non-specialist evaluated the size of the CNV perfused with FD2000 in a blinded manner, as described above. Sixteen spots per group (four spots per rat and four rats per group) were selected for statistical analysis. Abnormal spots due to excessive hemorrhage were excluded. The experiments were repeated twice to evaluate the reproducibility of the results.

### Statistical analysis

Experimental data are presented as means ± standard deviations. Statistical significance was assessed with BellCurve for Excel (Social Survey Research Information, Tokyo, Japan), using the unpaired *t*-test for normally distributed isolated pairs. *P* < 0.05 (*) or <0.01 (**) denoted statistically significant differences.

## Results

### Device fabrication

Figure 1 shows the configuration of the PDMS capsule device for transscleral drug delivery. The drug formulation composed of gelatin was sandwiched between a porous PDMS sheet and a non-porous PDMS sheet. The drug passed through the porous PDMS sheet, thus achieving one-way drug release. Therefore, the releasing side should be placed on the sclera during implantation. The flexibility of the device enabled easy handling during implantation.

### SEM observation

**Fig. 2:**
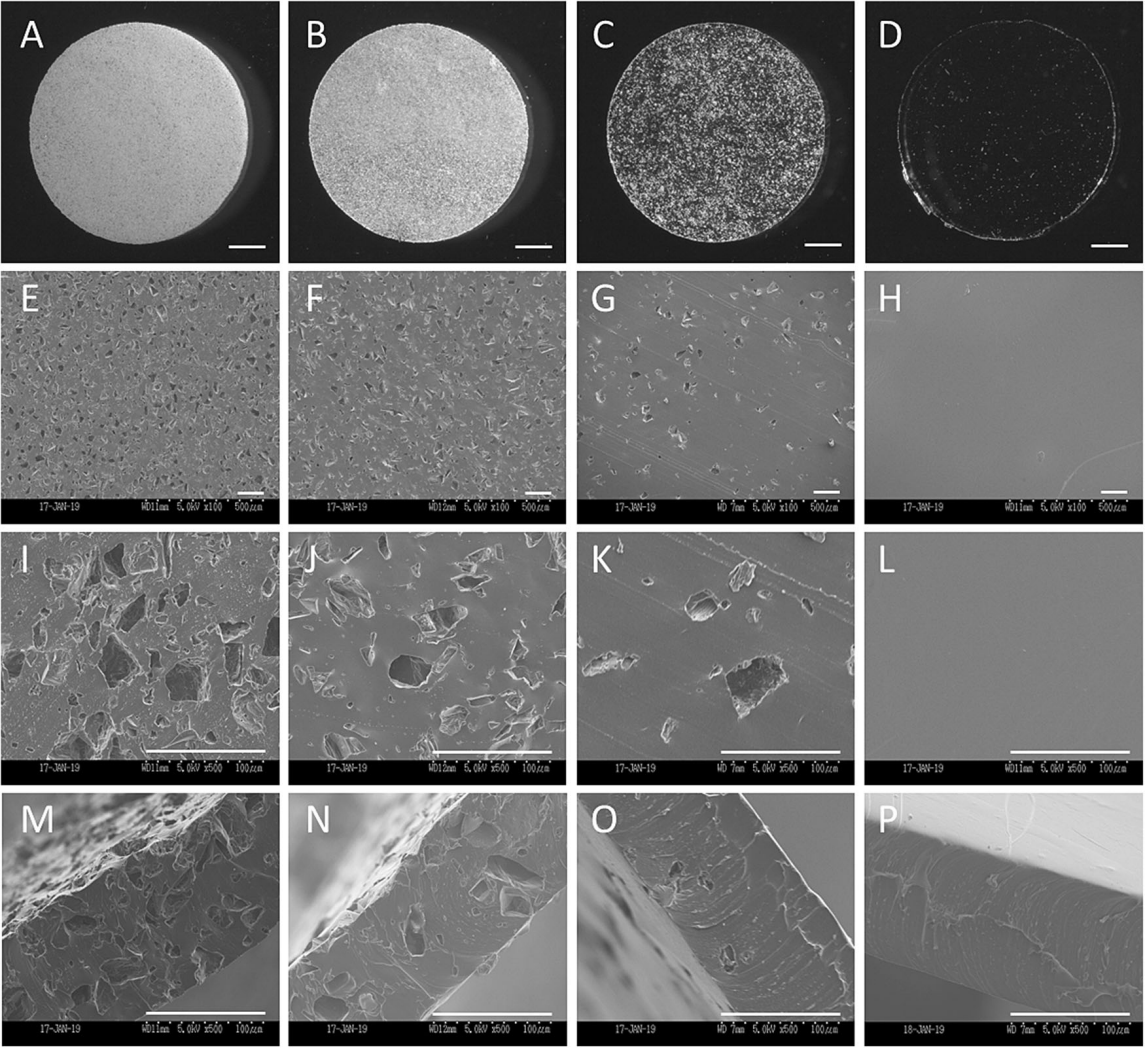
Representative images of the surface of the porous PDMS sheet with NaCl particle concentration of (**A**) 0.8, (**B**) 0.5, (**C**) 0.1, and (**D**) 0 g/mL. Bars: 1 mm. Representative scanning electron microscopic images of the surface of the porous PDMS sheet with NaCl particle concentration of (**E**, **I**) 0.8, (**F**, **J**) 0.5, (**G**, **K**) 0.1, and (**H**, **L**) 0 g/mL at ×100 (**E**–**H**) and ×500 (**I**–**L**) magnification. Cross-sectional images of the porous PDMS sheet with NaCl particle concentration of (**M**) 0.8, (**N**) 0.5, (**O**) 0.1, and (**P**) 0 g/mL. Bars: 100 μm. PDMS poly(dimethylsiloxane)

Figure 2 shows the SEM images of the surface and cross-section of porous PDMS sheets. The color of the sheets gradually became white in response to the increasing concentration of salt particles (Fig. 2A–D). Gross SEM images revealed that the pore density derived from leached salt particles is almost proportional to the concentration of those particles (Fig. 2E–H). Magnified images revealed that the pore diameter almost matched that of the salt particles (Fig. 2I–L). Cross-sectional images indicated the complete leaching out of salt particles and presence of interconnected pores (Fig. 2M–P).

### In vitro release of FITC-albumin and ranibizumab

**Fig. 3:**
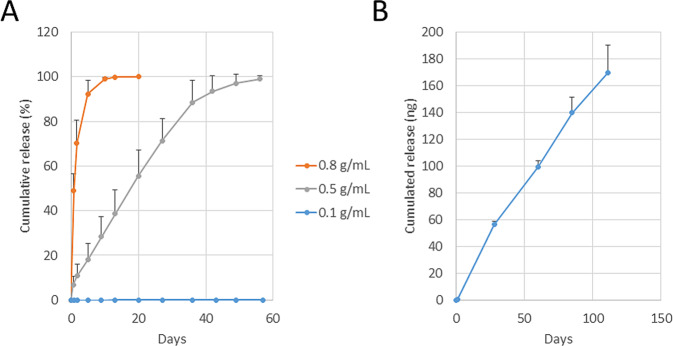
**A** In vitro release profile of FITC-albumin released from the porous PDMS sheet with NaCl particle concentration of 0.8, 0.5, and 0.1 g/mL. **B** In vitro release profile of ranibizumab released from the porous PDMS sheet with NaCl particle concentration of 0.5 g/mL. Data points are the mean ± SD; *n* = 4. FITC fluorescein isothiocyanate, PDMS poly(dimethylsiloxane), SD standard deviation

Figure 3 showed the release profile of FITC-albumin from the PDMS capsule. The PDMS capsule with a porous sheet containing salt at a concentration of 0.8 mg/mL showed burst release in the first week. In contrast, the capsule with a porous sheet containing salt at a concentration of 0.1 mg/mL exhibited slower release (Fig. 3A). The ranibizumab-loaded capsule with a porous sheet containing salt at a concentration of 0.5 mg/mL was characterized by constant release for 16 weeks; the average release rate was 1.5 ng/day (Fig. 3B).

### Flat mount for evaluation of the CNV area

**Fig. 4:**
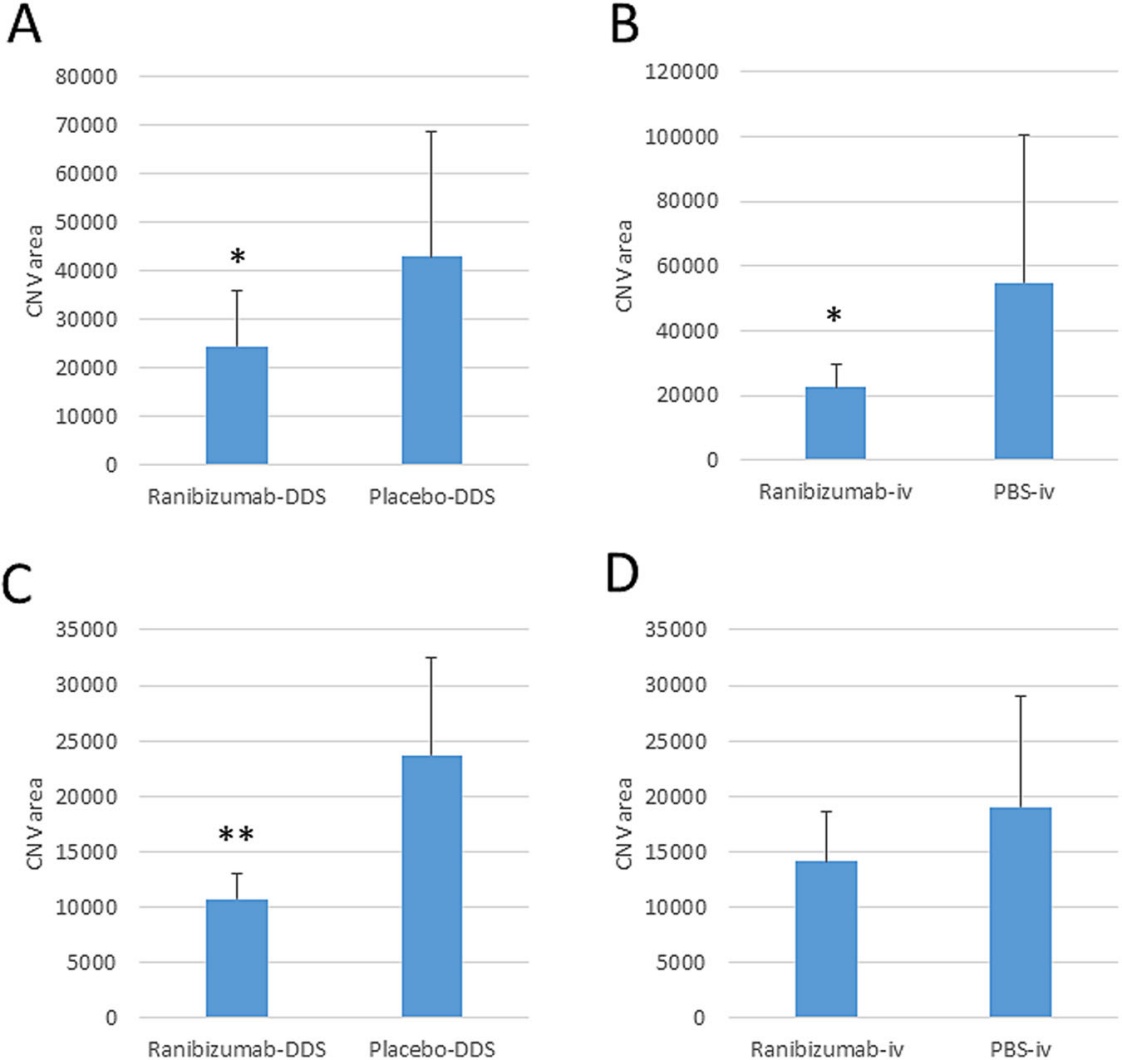
Evaluation of the CNV area at (**A**, **B**) 8 and (**C**, **D**) 18 weeks after treatment with (**A**, **C**) ranibizumab-DDS and (**B**, **D**) monthly intravitreal injections of ranibizumab. Data are the mean ± SD; *n* = 16. *P < 0.05; **P < 0.01 (*t*-test). CNV choroidal neovascularization, DDS drug delivery system, iv intravitreal injection, PBS phosphate buffered saline, SD standard deviation

Figure 4 shows the flat mount examination for the evaluation of the CNV area. At 8 and 18 weeks after implantation, the CNV area in the group treated with the ranibizumab-DDS was significantly smaller versus that observed in the group treated with the placebo-DDS. In the group treated with intravitreal injections of ranibizumab, a significantly smaller CNV was observed at 8 weeks after treatment; nevertheless, this effect was not observed at 18 weeks.

## Discussion

In this study, we fabricated a porous PDMS sheet with various pore densities. Moreover, we showed that the release of macromolecules, including albumin and ranibizumab, could be controlled by the pore density. We used a conventional salt-leaching method to produce pores. Initially, the salt was not leached out from the PDMS pellet. It was expected that the salt particles were wrapped inside the PDMS and not exposed on the surface. Therefore, the pellet was thinly sliced to expose the salt on the sliced surface. Subsequently, the slices were soaked in water, resulting in successful leaching out of the salt particles. Observation through SEM clearly showed the successful fabrication of the porous structure in the PDMS sheets, and the porous density was controlled by altering the concentration of salt. The moderate interconnected porous structure at the salt concentration of 0.5 g/mL could contribute to the sustained release of albumin and ranibizumab. The porous structure is useful in controlling the release rate of macromolecules. Currently, PDS is successfully used for the continuous delivery of ranibizumab for 6 months in clinical practice [14]. The system comprises a silicone body, septum port to inject the drug, and porous titanium as the element for the control of release [15]. In addition, electrospinning is a promising technique for the production of highly porous membranes. Augustine et al. reported the use of a electrospun porous poly(vinyl alcohol)/poly(lactic acid) membrane loaded with a connective tissue growth factor for diabetic wound healing [16]. Recently, biologics (e.g., antibodies and oligonucleotide therapeutics) revolutionized the approach to the treatment of disease. These new modalities have higher molecular weight than traditional small molecules. Thus, our porous PDMS membrane system would be a candidate for the controlled release of these molecules.

FITC-albumin was used as a model drug because its molecular weight was similar to that of ranibizumab. The release rate of FITC-albumin was controlled by adjusting the pore density of the porous PDMS sheet. The sheet containing salt at the concentration of 0.5 g/mL showed a long-term controlled-release profile. Hence, it was selected for the release of ranibizumab. We have reported the release of ranibizumab from a biodegradable gelatin/chitosan sheet [12]. In a biodegradable release system, initial burst release was observed due to the rapid diffusion of drug from the surface of the material. In this study, use of a porous PDMS sheet prevented the initial burst and provided constant release for 18 weeks. Thus, the non-biodegradable controlled-release membrane system offered an advantage with regard to the controlled release of ranibizumab. Our CNV experiments demonstrated the long-term efficacy of the ranibizumab-releasing PDMS capsule via the transscleral administration route. It has been reported that the half maximal inhibitory concentration (IC_50_) of ranibizumab for the inhibition of VEGF-induced endothelial cell proliferation was 0.4–1.2 nM (19.3–58.0 ng/mL) [17]. The results of the in vitro release analysis of the ranibizumab-DDS indicated that >30 ng/mL of drug per day (1.5 ng/day divided by 50 μL of rat vitreous volume) was released during the 16-week incubation. This rate was several folds lower than the IC_50_. Our previous results showed drug accumulation around the RPE in rats after transscleral drug delivery [18]. These findings suggested that accumulation of ranibizumab around the RPE and retina was possible. Thus, the accumulation of ranibizumab may affect the long-term efficacy of the treatment. Further studies using larger animals are warranted to evaluate the drug distribution in the eye and the applicability of the devices to humans.

An injectable sheet offers the advantage of less invasiveness compared with the reservoir-type device previously used [18]. The previously used device required surgical implantation on the sclera and an incision (length: 4 mm) of the conjunctiva. In contrast, the flexible PDMS sheet could be loaded into the subtenon space by injection using a syringe needle, similar to a previously reported foldable sheet-type device [19]. This approach eliminates the requirement for an incision of the conjunctiva. There were no abnormal findings on the sclera related to the long-term implantation of PDMS sheet. Nevertheless, it is necessary to evaluate potential reactions of the animals to the presence of this material (e.g., immune response and scar formation around the device). In the future, we plan to evaluate the injectability of the PDMS sheet in rabbits and further determine the response of animals to this material. Moreover, we intend to investigate the drug distribution in the retina after transscleral sheet injection.

## Conclusion

We produced a novel controlled-release device fabricated by salt-leaching of a micro-sectioned PDMS sheet containing salt microparticles. The pore densities could be adjusted by the concentration of salt and the release rate of albumin could be adjusted based on the pore density of the porous PDMS sheet. Using this device, ranibizumab could be released in a sustained-release manner and suppress CNV via the less invasive transscleral route compared with intravitreal injections. Prolonged sustained release could reduce the frequency of drug administration, thereby reducing the occurrence of side effects associated with repeated injections.
